# Genetic diversity of the obligate intracellular bacterium *Chlamydophila pneumoniae *by genome-wide analysis of single nucleotide polymorphisms: evidence for highly clonal population structure

**DOI:** 10.1186/1471-2164-8-355

**Published:** 2007-10-04

**Authors:** Thomas Rattei, Stephan Ott, Michaela Gutacker, Jan Rupp, Matthias Maass, Stefan Schreiber, Werner Solbach, Thierry Wirth, Jens Gieffers

**Affiliations:** 1 Department of Genome-Oriented Bioinformatics, Technische Universität München, Wissenschaftszentrum Weihenstephan, Am Forum 1, 85354 Freising, Germany; 2 Institute of Clinical Molecular Biology, Christian-Albrechts-University Kiel, Schittenhelnstrasse 12, 24105 Kiel, Germany; 3 Instituto Cantonale di Microbiologia, Via Mirasole 22, 6501 Bellinzona, Switzerland; 4 Institute of Medical Microbiology and Hygiene, University of Lübeck, Ratzeburger Allee 160, 23538 Lübeck, Germany; 5 Institute of Medical Microbiology, Hygiene and Infectious Diseases, University Hospital Salzburg, Muellner Hauptstrasse 48, 5020 Salzburg, Austria; 6 Department of Biology, Lehrstuhl für Zoologie und Evolutionsbiologie, University Konstanz, Universitätstrasse 10, 78457 Germany

## Abstract

**Background:**

*Chlamydophila pneumoniae *is an obligate intracellular bacterium that replicates in a biphasic life cycle within eukaryotic host cells. Four published genomes revealed an identity of > 99 %. This remarkable finding raised questions about the existence of distinguishable genotypes in correlation with geographical and anatomical origin.

**Results:**

We studied the genetic diversity of *C. pneumoniae *by analysing synonymous single nucleotide polymorphisms (sSNPs) that are under reduced selection pressure. We conducted an in silico analysis of the four sequenced genomes, chose 232 representative sSNPs and analysed the loci in 38 *C. pneumoniae *isolates. We identified 15 different genotypes that were separated in four major clusters. Clusters were not associated with anatomical or geographical origin. However, animal lineages are basal on the *C. pneumomiae *phylogeny, suggesting a recent transmission to humans through successive bottlenecks some 150,000 years ago. A lack of detectable variation in 17 isolates emphasizes the extraordinary genetic conservation of this species and the high clonality of the population. Moreover, the largest cluster, which encompasses 80% of all analysed strains, is an extremely young clade, that went through an important population expansion some 3,300 years ago.

**Conclusion:**

sSNPs have proven useful as a sensitive marker to gain new insights into genetic diversity, population structure and evolutionary history of *C. pneumoniae*.

## Background

The order *Chlamydiales *evolved from their free-living ancestors about 500–1000 million years ago (mya) [1], establishing their intracellular life in lower eukaryotes. Their life cycle consists of infectious elementary bodies and intracellularly replicative reticulate bodies (for recent review see e.g. [2]. The divergence of *C. trachomatis *and *C. pneumoniae *50–200 mya proceeds the appearance of *Homo sapiens *and, thus, each lineage possessed sufficient biological potential to exploit new hosts [1] Gene acquisition by horizontal gene transfer (HGT) was not a driving force in the evolution of the *Chlamydiales*; less than 1 % of the total gene number was estimated to result from HGT [3]. Instead, while adapting to the homeostatic niche within their specific hosts, all Chlamydiaceae species reduced their genome size to little more than 1 Mb. At least 80 % of the genes in any sequenced *Chlamydiaceae *genome represent orthologs with other Chlamydiaceae species [4-6]. Despite this high level of functional conservation, chlamydiae differ in their host spectrum, tissue tropism and spectrum of host diseases resulting from the infection.

*Chlamydophila pneumoniae *deserves medical attention as an important respiratory pathogen causing about 10 % of community acquired pneumonias and upper respiratory tract infections like bronchitis, pharyngitis and sinusitis [7,8]. Virtually every person is believed to be infected at least once during their lifetime. Serological evidence indicates that infections start to occur during childhood and 50 – 60 % of the population has been exposed by 20 years of age [9]. Viable chlamydiae have been isolated from atherosclerotic plaques, implicating a causal role in the development of atherosclerosis [10]. Blood monocytes are believed to be the vector system within the systemic circulation [11]. Moreover, *C. pneumoniae *has been isolated in the brain and is associated with Alzheimer's disease and Multiple Sclerosis [12,13].

In contrast to *C. trachomatis*, no biovars or pathotypes have been described so far for *C. pneumoniae*, despite of the broad range of disease associations and sites of infection. The genomic sequences of the four isolates AR-39, CWL-029, J138 and TW-183 show a remarkable level of gene conservation and synteny. Overall identity between isolates is > 99% [4-6]. The genomes of the four isolates comprise an equivalent set of coding sequences and differ only by a small number of insertions and deletions and several single nucleotide polymorphisms (SNPs). Analyses of repeated sequences suggested that *C. pneumoniae *has the highest potential for recombination among fully sequenced Chlamydiaceae and that the majority of recombination hotspots of the *C. pneumoniae *genome are concentrated in a family of polymorphic proteins [14]. The high degree of conservation is in line with the low rate of HGT as well as a low frequency of genome rearrangements within the species, owing to the unique chlamydial biology and ecological isolation within the intracellular niche. Therefore, point mutations, fixed in the population and accumulating with time, are the major source of genetic variability in *C. pneumoniae *hence SNPs are among the most sensitive phylogenetic markers to reconstruct the evolutionary history of this species. SNPs occur as two classes of substitutions within genes, referred to as synonymous and non-synonymous SNPs (sSNPs; nsSNPs). nsSNPs result in amino acid replacement and are targets of evolutionary selection. sSNPs make a difference with respect to the codon usage but do not alter the protein sequence and are therefore evolutionary almost neutral. This qualifies sSNPs as a valuable target for population genetic studies. SNPs have been successfully used to reconstruct the phylogeny of the *M. tuberculosis *complex [15] and *B. anthracis *isolates [16]. In the present study, we analysed SNPs of *C. pneumoniae *to answer questions about the homology of geographically and anatomically different isolates and the existence of distinguishable genotypes. We first conducted an in silico analysis of sSNPs and nsSNPs of the four sequenced *C. pneumoniae *isolates. To reduce the potential ascertainment bias caused by deriving the SNP events from a limited set of only four completely sequenced genomes, the SNPs selected for sequencing should be as independent as possible. Compared to non-coding and non-synonymous SNPs, synonymous SNPs provide evolutionary almost neutral, abundant and equally distributed sequence markers in the *C. pneumoniae *genomes. We therefore chose a representative subset of 232 sSNPs and analysed the loci in 38 *C. pneumoniae *isolates. As chlamydiae are difficult to propagate the available collection is the largest that has even been analysed. By this approach, we were able to gain new insights into the genetic diversity, the population structure and evolutionary history of *C. pneumoniae*.

## Results

### SNP-analysis of the reference strains

**Figure 1:**
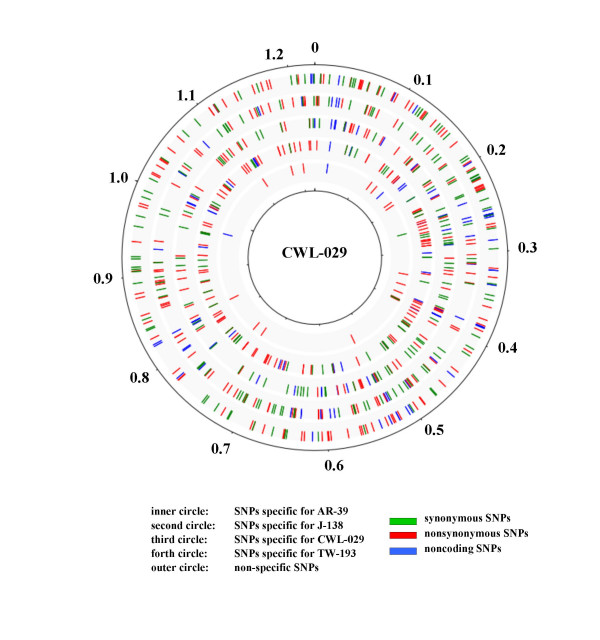
Distribution of SNPs within the genome. SNPs are equally distributed around the genome; no mutational hot-spot could be identified.

Comparative genome analysis of the four sequenced reference strains revealed a total of 688 SNPs within the 1.2 mega base genome. The ratio of transitions:transversions has been determined at 2.7:1 for synonymous SNPs, 1.4:1 for nonsynonymous SNPs, 1: 1.07 for SNPs in intergenic regions and 1:2 for RNA genes. SNPs of all classes were homogeneously distributed over the whole genome (figure 1). No mutational hot spot could be identified. The number of SNPs per gene did not exceed 6. There was no functional class of genes that accumulated more SNPs than average (data not shown). The individual genes that contains SNPs were provided in additional file 1. Table 1 specifies the number of different SNPs identifying certain isolates. J-138 was the most unique isolate. The number of nsSNPs exceeded that of sSNPs by a ratio of 1.45:1. Given the same mutation probability for all positions and the absence of any selection pressure, we calculated a nsSNP/sSNP ratio of 3.4:1 being the null hypothesis.

**Table 1:** Origin of the *C. pneumoniae *isolates

**Isolate**	**Site of Isolation**	**Geographical Area**	**Source (Laboratory)**
Cluster I			
DC-9	frog (*Cryptohylax gresshoffi*), liver	Central African Republic	Sachse
Koala	Koala	Australia	Timms
Cluster II			
TW-183	conjunctiva	Taiwan	Grayston/ATCC VR-2282
A-03	coronary artery	USA/Louisville	Summersgill/ATCC CCL23
UZG1	respiratory tract	The Netherlands	Ossewaarde/CDC
IOL-207	conjunctiva	Iran	Nicolini/CDC
Cluster III			
CM-1	sputum	USA/Atlanta	Black/ATCC VR-1360
YK-41	nasopharynx	Japan/Hiroshima	Kanamoto/CDC
AR-39	pharynx	USA/Seattle	Grayston/ATCC 53592
J-138	respiratory tract	Japan	(in silico)
Cluster IV			
Wien-2	femoral artery	Austria/Vienna	Apfalter
Wien-3	infrarenal aneurysm	Austria/Vienna	Apfalter
MUL-2216	bronchoalveolar lavage	Germany/Lübeck	Maaß
MUL-250	respiratory tract	Germany/Lübeck	Maaß
MUL-2076	bronchoalveolar lavage	Germany/Lübeck	Maaß
MUL-2090	bronchoalveolar lavage	Germany/Lübeck	Maaß
PB-1	peripheral blood monocytes	Germany/Lübeck	Maaß
PB-2	peripheral blood monocytes	Germany/Lübeck	Maaß
PB-3	peripheral blood monocytes	Germany/Lübeck	Maaß
CV-14	coronary artery	Germany/Mainz	Maaß
CV-15	coronary artery	Germany/Mainz	Maaß
CV-16	coronary artery	Germany/Mainz	Maaß
CV-17	coronary artery	Germany/Mainz	Maaß
CV-18	coronary artery	Germany/Mainz	Maaß
CWL-011	throat	USA/Atlanta	Black/CDC
CWL-029	throat	USA/Atlanta	Black/ATCC VR-1310
AL-1	respiratory tract	Sweden/Umea	Boman
W-5	respiratory tract	USA/Madison	MacDonald/CDC
U-1360	respiratory tract	Sweden/Umea	Boman
U-1271	respiratory tract	Sweden/Umea	Boman
U-1273	respiratory tract	Sweden/Umea	Boman
W-6	respiratory tract	USA/Madison	MacDonald/CDC
T-45	respiratory tract	Sweden/Umea	Boman
CWL-050	throat	USA/Atlanta	Black/CDC
Panola	respiratory tract	Finnland	Saikku
Helsinki-12	respiratory tract	Finnland	Saikku
Kajaani-6	respiratory tract	Finnland	Saikku
Kajaani-7	respiratory tract	Finnland	Saikku

### Phylogeny of *C. pneumoniae*

**Figure 2:**
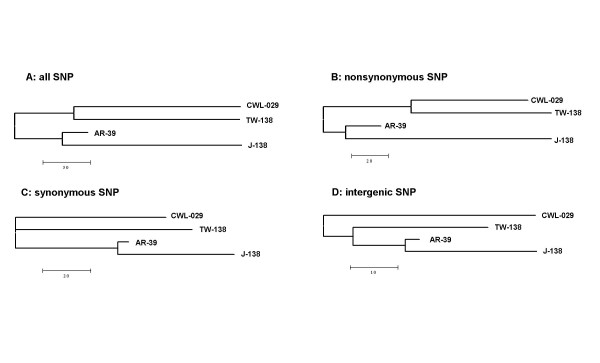
Phylogeny of *C. pneumoniae *reference isolates based on different SNP types (see table 2). Distance calculated as number of SNPs. Analysis revealed a phylogenetic proximity between CWL-029 and TW-183, and AR-39 and J-138, respectively. The topology of the tree was not significantly altered when sSNP, nsSNP or SNP of intergenic regions were analysed separately. Only sSNPs were not able to resolve the common branch of CWL-029 and TW-183.

**Figure 3:**
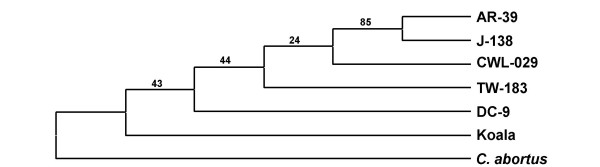
*C. pneumoniae *consensus tree topology based on multiple and independent phylogenetic reconstructions (Neighbor-joining, Minimum Evolution and Parsimony) with *C. abortus *as an outgoup. At least one representative of the clusters of figure 4 are analysed. Animal strains are basal and the human strains are derived, suggesting a zoonosis scenario for *C. pneumoniae*.

A comparison of the evolutionary relation between the four isolates revealed a phylogenetic proximity between CWL-029 and TW-183, and AR-39 and J-138, respectively (figure 2A). The topology of this evolutionary tree was not significantly altered when sSNP, nsSNP or SNP of intergenic regions were analysed separately (figure 2B–D). sSNPs were not able to resolve the common branch of CWL-029 and TW-183 (figure 2C). In a second attempt, we tried to find a valuable outgroup in order to unravel the tree topology of the *C. pneumoniae *complex. From multiple blast search it appeared that *C. abortus *is the closest published genome to *C. pneumoniae*, though being rather distant (20% divergence). Using different phylogenetic reconstructions (neighbor-joining, minimum evolution and maximum parsimony) we obtained the same tree topologies where the animal isolates are basal and the human isolates are derived, suggesting a zoonosis scenario for *C. pneumoniae *(figure 3). However, these results have to be taken with caution, since this topology was only weakly supported (< 70% bootstrap values) with the exception of the AR-39-J-138 clade. Due to the limited number of only two available animal isolates, the effect of long-branch attraction (LBA) cannot be excluded for this part of the tree. Nevertheless, the consistent topology derived by parsimony and distance methods LBA seems not to be the most probable explanation.

**Figure 4:**
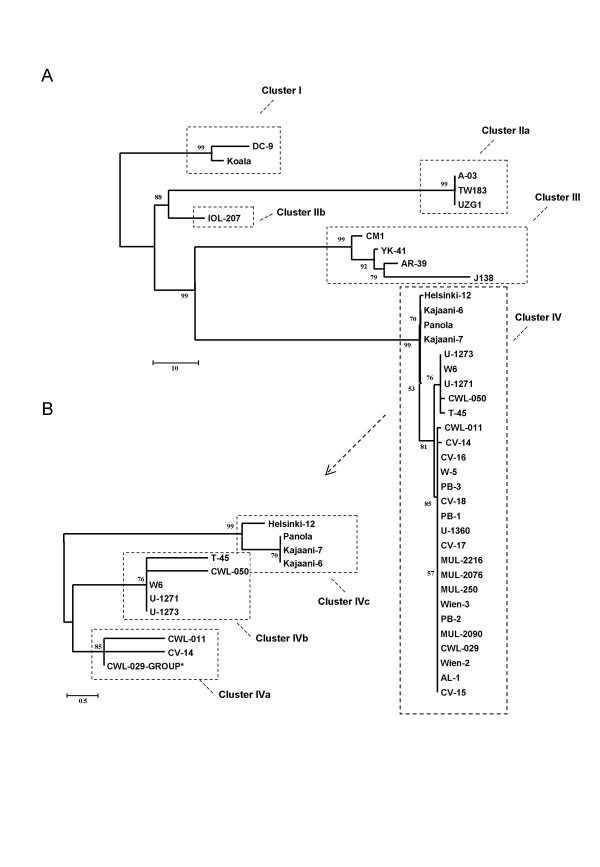
Phylogeny of 38 *C. pneumoniae *isolates based on 232 sSNPs using the neighbor-joining method with 1000 bootstrap replicates and distances calculated using the number of different SNPs. The marked section of panel A is enlarged in a separate subtree in panel B (* CWL-029-Group: CWL-029; CV-15 to -18; PB-1 to -3; MUL-250, -2076, -2090, -2216; Wien-2, -3; Al-1, W-5, U-1360). Four separate cluster can be distinguished.

The phylogeny of 38 *C. pneumoniae *isolates based on 232 sSNPs is shown in figure 4. The tree shows four distinguishable clusters. The animal isolates of the African frog (DC-9) and Australian Koala form one cluster. The Iranian ocular isolate IOL-207 and the three indistinguishable isolates TW-183 (conjunctival, Taiwan), A-03 (coronary artery, USA) and UZG1 (respiratory tract, The Netherlands) have likely evolved from a common ancestor and form a second cluster. Cluster III is represented by the two Japanese isolates J-138 and YK-41 and two U.S. isolates AR-39 and CM-1. Cluster IV is the biggest and contains 28 isolates. Isolates within this cluster differ in 5 SNPs at most. The four Finnish isolates (Helsinki-12, Kajaani-6, Kajaani-7, Panola) form a distinct subcluster. Within the remaining closely related isolates, 17 isolates are identical by SNP analysis and are termed the CWL-029 group. This group contains mainly respiratory and cardiovascular isolates as well as those from peripheral blood monocytes. All isolates except the U.S. respiratory isolates CWL-029 and W-5 are of European origin. One cardiovascular isolate (CV-14) differs from the other European cardiovascular isolates by one SNP. The U.S. isolates CWL-011, CWL-050 and W-6 can be differentiated from the CWL-029 group by one to two isolate specific SNPs. Five Swedish isolates were included into the study. They all are closely related, but only AL-1 and U-1360 are located indistinguishable in the CWL-029 group. T-45 differs by one SNP from U-1273. U-1273 and U-1271, which are identical to each other, differ by two additional SNPs from the CWL-029 group. The split between the main *C. pneumoniae *lineages is rather young; for example the mean number of mutations between the ancestral cluster I (frog = koala) and cluster IV is 95.2 (± 7.4) mutations, this translates into 0.14% sequence divergence, which suggests that these two lineages separated some 150,000 years ago. This analysis is based on the *E. coli *clock rate. We are aware that the evolutionary clock of obligate intracellular organisms might run faster; that would even reduce the calculated age of clades.

### Demographic history

**Figure 5:**
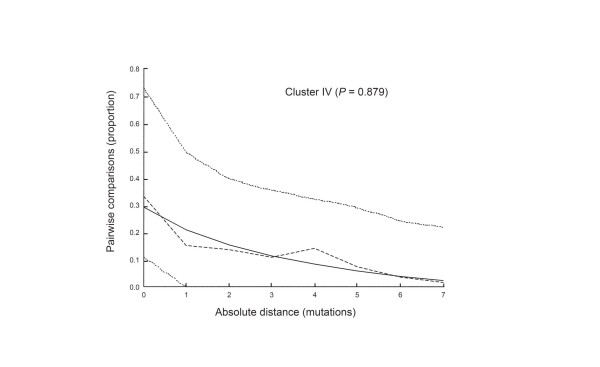
Mismatch distribution of cluster IV strains for the concatenated sequences (sSNPs). These curves represent the frequency distribution of pairwise differences. The dashed plot correspond to the observed data; the smoth curve corresponds to the sudden expansion model and the punctuated lines represent the upper and lower bounds of the 95% confidence interval on this model. *P *value represents the probability that the raggedness of the simulated data set is equal to or greater than the observed data set. The major demographic extension occurred 2 mutations ago for the entire concatenated genes (70 kb). The latter translates into 0.003% sequence divergence, which suggests that the maximum population expansion occurred about 3,300 years (700 to 7,200 years) ago.

We computed frequencies of pairwise differences between the large cluster IV haplotypes (the mismatch distribution of the raw data) to evaluate the hypothesis of recent population growth in *C. pneumomiae *(figure 5). The distribution of nucleotide differences between pairs of haplotypes shows the typical unimodal shape of a population having passed through a recent demographic expansion [17] and fits a sudden expansion model (the model could not be rejected, *P *= 0.879). Based on the stepwise expansion model, the effective population size of this lineage increased by a factor of 1,000 (θ_0 _= 0.002 and θ_1 _= 2.437). Assuming a synonymous mutation rate (μ) of 9 × 10^-9 ^per nucleotide per year and a τ-value of 4.967 (95% interval confidence 1.427–10.967), the expansion event took place about 3,300 years ago.

### Genotyping of *C. pneumoniae*

SNP data revealed to be useful to genotype new *C. pneumoniae *isolates and to allocate them to the described clusters. A subset of sSNPs was defined that discriminates between the genotypes. Additional file 2 gives an algorithm for the identification of different *C. pneumoniae *isolates. A total of 12 different SNPs (four to five SNPs per isolate) are sufficient to identify these isolates or to relate a new isolate to the characterized ones.

## Discussion

The evolutionary tree of the 38 *C. pneumoniae *isolates based on 232 sSNPs displays the genetic variability of the species. The topology ot the phylogenetic tree of the four reference isolates (figure 2) is reflected within the tree. There are four major clusters within the tree and 15 different *C. pneumoniae *genotypes can be defined by the sSNP analysis.

Of all reported studies, this is the highest discriminative analysis of genetic diversity in *C. pneumoniae *and sSNPs have proven useful to differentiate between *C. pneumoniae *isolates. Nevertheless, it is most intriguing that even with this sensitive marker we were not able to differentiate 17 out of 38 isolates that originated from different European and American origins and anatomical localisations. This is evidence for a highly clonal population structure and advocates for a recent origin of this bacterial species. Results are in line with the expected widespread homologous recombination in *C. pneumoniae*.

The genetic clusters do not separate between geographic or anatomical origin of the isolates, even if the Finnish isolates form a distinct subcluster. Isolates that infect the blood or the vasculature cannot be separated from respiratory isolates, thus we hypothesize that they are not genetically different lineages and are rather young. We calculated that the expansion event in cluster IV took place about 3.300 years ago or even more recently assuming a faster clock rate in intracellular bacteriae. Nevertheless, vascular and PBMC isolates have been shown to possess a single copy of the *tyr*P gene while respiratory isolates have multiple copies. A single *tyr*P copy only has been proposed to improve the propensity for vascular infection and persistence [18]. We conclude that the multiplication of the *tyr*P gene might have occurred after the emergence of the genotypes defined by SNPs. Moreover, the identiy of vascular and respirotory isolates in the sSNP analysis does not exclude the possibility of different pathotypes, as sSNPs have no phenotypic correlate. To further study genotype-phenotype correlation, non-synonymous polymorphisms would be more suitable. Given the clonal population structure demonstrated in this study, we assume that polymorphisms defining potential pathotypes can be discovered only by the most sensitive techniques, e.g. the sequence analysis of the whole genome.

We are aware that this sSNP analysis only provides limited information on the species phylogeny and may be biased by the ascertainment of the sequenced SNPs. The SNPs we chose for analysis were identified by four reference isolates. This approach includes an inherent bias, as additional SNPs of other isolates remain unconsidered. The most likely effect of this bias could be a branch collapse and the underestimation of the evolutionary distance. However, we are convinced that the impact of this bias is limited by the fact that the reference isolates come from distant locations, and, thus, represent different genotypic lineages. TW-183 is an ocular strain obtained in Taiwan in 1965, AR-39 was isolated from a patient with acute respiratory symptoms in Seattle, USA in 1983. CWL-029 was isolated from a pneumonia patient in Atlanta, USA in 1988. J-138 was obtained from a boy with pharyngitis in Japan in 1994. This assumption is supported by the fact that each identified cluster, with the exception of the animal isolates, includes one reference isolate and no distant outgroups occurred. Additionally, when we sequenced about 8 % of the genome, we did not identify novel SNPs in human strains that were absent in the reference strains. In contrast, we detected 43 new sSNPs between animal and human isolates. Extrapolating from the 0.1 mega bases sequenced, one can speculate, that about 500 additional sSNPs could be found in the whole genome. Thus, we believe that the evolutionary distance between animal and human strains is highly underestimated in this tree (figure 4).

The major phylogenetic finding is that animal *C. pneumoniae *isolates from African frog and Australian koala cluster closely together and differ distinctly from human isolates. Highest homologies outside the species are found with *C. abortus*, a ruminant pathogen that also possess the ability to infect humans. Further analysis showed that the animal isolates are basal and the human isolates are derived. We, thus, hypothesized that *C. pneumoniae *evolved from an animal pathogen and the *C. pneumoniae *animal isolates are the ancestors of human isolates. Two previous studies analysing the 16S rRNA gene, the 16S/23S intergenic spacer and domain I of the 23S gene supports this hypothesis: A *C. pneumoniae *isolate from a horse clustered different from human isolates that were derived [19,20]. The split between the lineages in our study occured in recent history, about 150,000 years ago. While spread and mixture of human isolates by migration and travel could explain their genetic similarity, it is extremely unlikely, that Australian koala and African frog were able to exchange the intracellular parasite. Thus, it is most intriguing that both isolates differ by only two SNPs despite the long period of evolutionary isolation. We interpret this as evidence for highly clonal population structure as a consequence of a very conserved genome. Additionally, we speculate that the transition into the human or animal population represents an evolutionary bottleneck that contributed to the clonal population structure. A similar evolutionary history was proposed for *M tuberculosis *based on the conservation of point mutations and deletions [21,22].

When we analysed the four sequenced genomes in silico, a first remarkable finding was that the abundance of nsSNPs exceeded that of sSNPs (ratio of 1.45:1). In an environment of strong selective pressure one could expect that the number of sSNPs would exceed the number of nsSNPs because the latter are purged from the population by purifying selection. To solve this apparent contradiction we determined the likelihood for a single nucleotide exchange to cause a nsSNP or sSNP for the CWL-029 genome. Absence of any selection would result in a ratio of 3.4:1 between nsSNPs and sSNPs. Thus, the reduced number of observed nsSNP in comparison to the theoretically expected one is evidence for strong selective pressure within the intracellular niche.

We further found that all SNPs are homogeneously distributed around the genome (figure 1). In this respect, *C. pneumoniae *differs from *C. trachomatis*, which shows a significant amount of genetic variation between serovars within the "plasticity zone" around the origin of replication [4,23]. No class of genes (e.g. outer membrane proteins) could be identified that showed more SNPs than the average (data not shown). This finding correlates with the low number of recent HGT events reported for the *C. pneumoniae *species [5].

## Conclusion

In summary, we have shown that sSNPs are a sensitive tool to reveal the genetic diversity and past demography of *C. pneumoniae *that remained undetected by other methods. Analysing a good portion of the worldwide available isolates, we were able to divide *C. pneumoniae *into different genotypes. Nevertheless, a lack of detectable variation in some isolates emphasizes the extraordinary genetic conservation of this species and the high clonality of the population. High selection pressure within the intracellular niche and an evolutionary bottleneck as a consequence of the adaptation to the human or animal host might have contributed to the stability of the genome.

## Methods

### Isolates

38 *C. pneumoniae *isolates of different geographical and anatomical origin were analysed. Table 2 specifies the anatomical site of isolation, the geographical origin and the source. Chlamydiae were cultivated on HeLa-229 cells as described previously [24]. They were purified by centrifugation on 30 % Urografin (Schering, Berlin, Germany). DNA was extracted by Nucleo Spin Tissue kit (MacheryNagel, Dueren, Germany). To avoid cross contamination, different isolates were manipulated in strict separation. We cannot exclude the possibility of cross contaminations in other laboratories. Nevertheless, as we obtained the isolates from various sources, single contaminations would not influence the overall conclusions of the study. The isolate collection was composed with regard to the highest possible geographical and anatomical diversity.

**Table 2:** SNPs unique for the indicated reference isolate(s)

	**Number of SNPs**		
**Isolate(s), identified by SNP **	**synonymous**	**non-synonymous**	**intergenic/rna**
CWL-029	61	63	52
AR-39	3	18	3
J138	47	112	29
TW-183	72	76	28
AR-39+CWL-029	0	0	0
AR-39+J-138	44	62	11
AR-39+TW-183	3	3	1
			
total	230	334	124

### In silico analysis of SNPs

To identify SNPs within the four sequenced isolates, we implemented a Java program which performed the tasks of calculating sequence alignments and extracting the SNPs from the alignments. The genomic sequences and annotations were downloaded from the Genbank database [25]. Starting from the CWL-029 genome and comparing it to the other genomes, syntenic protein coding regions, RNA genes and intergenic regions were identified by Smith-Waterman alignments. The gap-free part of the alignment with the best hit was used for SNP identification, if the fraction of mismatches did not exceed 5%. SNPs were identified from these alignments and classified according to their position on the CWL-029 genome to estimate their specificity. Protein coding SNPs were additionally classified into synonymous and non-synonymous SNPs. The distribution of SNPs around the genome (figure 1) was displayed by GenomeDiagram [26]. The phylogeny of the reference strains was conducted with MEGA 3.1 [27] using the neighbour-joining method with 1000 bootstrap replicates, and distances calculated using the number of different SNPs. The position of polymorphic genes and gene products within metabolic pathways were demonstrated by KEGG pathway assignments to the genes of the CWL-029 genome. They were manually extracted from the pathway maps at the KEGG website [28]. In order to estimate the odds that, for the CWL-029 genome, a single nucleotide mutation would result in a sSNPs or nsSNPs, the number of synonymous and nonsynonymous sites in all coding DNA sequences of the CWL-029 genomes was calculated. Based on the bacterial genetic code we classified each coding nucleotide by the consequence of the three possible mutations. Thus, we identified 842596 sites at which nsSNPs and 245642 sites at which sSNPs could occur.

### PCR and sequencing

According to the locations of sSNPs 189 loci evenly distributed around the whole genome were chosen for further analysis. The adjacent up- and downstream region (approx. 500 bp) of each SNP was amplified by PCR (primers provided in additional file 3) according to common protocols. 8 μl of the PCR product were digested with 0.3 U SAP (shrimp alkaline phosphatase) and 1.5 U ExoI (both Amersham Biosciences, Freiburg, Germany) for 15 min. The reaction was stopped by heating for 15 min at 72°C. The sequencing reaction was performed using 1 μl of ABI PRISM™ BigDye™ (Applied Biosystems, Foster City, USA), a 1.6 μM concentration of each sequencing primer (Eurogentec, Seraing, Belgium) and 2 μl of digested PCR product. The reaction conditions were 96°C for 10 s followed by 25 cycles of 95°C for 1 min, and 60°C for 175 s.

### Data and phylogenetic analysis

Chromatograms were analysed with Bio Edit [29] and SNPs were analysed using a Local Blast comparison with the CWL-029 isolate [30]. An additional 43 sSNPs were identified within the sequenced genome fragments (~0.1 mega bases). The SNP data of totally 232 loci were concatenated, resulting in one character string (nucleotide sequence) for each strain. The raw data are provided as additional file 4. Concatenated alignments are given as additional file 5. Phylogenetic analyses were conducted with MEGA 3.1 [27] using the neighbour-joining method with 1000 bootstrap replicates and distances calculated using the number of different SNPs. In order to root the *C. pneumoniae *phylogeny, we blasted 50 gene fragments that we had already sequenced from the DC-9 and Koala isolates. The highest homologies were obtained for *Chlamydophila abortus*, an endemic ruminant bacteria that colonizes the placenta. Using this approach we finally obtained a 2 kb alignment of orthologous gene fragments. The dating of different lineages split was calculated based on an *E. coli *molecular clock rate. Comparisons of homologous protein-coding regions from *E. coli *and *S. enterica *indicated an average rate of sequence divergence at synonymous sites of 0.9% per millions years, a value extrapolated from a 120–160 million years divergence time [31,32].

### Demographic inferences

To determine whether some *C. pneumoniae *populations underwent recent population expansions, we calculated mismatch distributions and compared these to predicted distributions from models of population expansion[33]. For expanding populations, we converted the parameter *tau *(τ; calculated from the mismatch distribution) to estimate the time of the expansion (*t*) using the equation τ = 2μ*t*, where μ is the neutral mutation rate for the locus. The confidence intervals of τ were calculated using a parametric bootstrap approach [34]. Mismatch distributions and τ were calculated in ARLEQUIN 3.0 [35].

## Abbreviations

sSNP: synonymous single nucleotide polymorphism

nsSNP: non-synonymous single nucleotide polymorphism

mya: million years ago

HGT: horizontal gene transfer

LBA: long branch attraction

PBMC: polymorphic blood mononuclear cells

## Competing interests

the author(s) declares that there are no competing interests.

## Authors' contributions

TR carried out all bioinformatics and drafted the manuscript. SO and SS carried out the PCR and sequencing. MG, WS and JR drafted the manuscript. MM isolated a majority of the isolates. TW carried out the phylogenetic analysis. JG conceived, designed and coordinated the study and drafted the manuscript. All authors read and approved the final manuscript.

## Supplementary Material

Additional file 1 Genes containing synonymous or non synonymous polymorphisms. This table list the genes and protein IDs containing synonymous or non synonymous polymorphisms as identified in this study. Click here for file

Additional file 2 Algorithm for identification of *C. pneumoniae *isolates by different SNPs. This figure allows for the identification of *C. pneumoniae *isolates by different SNPs. Numbers indicate SNP positions within the CWL-029 genome. The first branches are identified by two SNPs. The primers for identification for the isolates can be identified in additional file 3. Click here for file

Additional file 3 Primers. This table lists the primers used for amplification of SNP containing fragments. Click here for file

Additional file 4 SNP analysis raw data. This data give the detected base for each isolate and each polymorphic locus. Click here for file

Additional file 5 Concatenated alignment for phylogenetic reconstruction. This file provides the concatenated alignment of the SNP sequence data which was used for phylogenetic reconstruction. Click here for file
