# Supplementary material for: Genetic diversity of the obligate intracellular bacterium Chlamydophila pneumoniae by genome-wide analysis of single nucleotide polymorphisms: evidence for highly clonal population structure
Source: BMC Genomics. 2007 Oct 4;8:355. doi: 10.1186/1471-2164-8-355 (PMC2092436; doi:10.1186/1471-2164-8-355)
Supplement: Additional file 2 — Algorithm for identification of C. pneumoniae isolates by different SNPs. This figure allows for the identification of C. pneumoniae isolates by different SNPs. Numbers indicate SNP positions within the CWL-029 genome. The first branches are identified by two SNPs. The primers for identification for the isolates can be identified in additional file 3. [file 1471-2164-8-355-S2.ppt]

## Slide 1
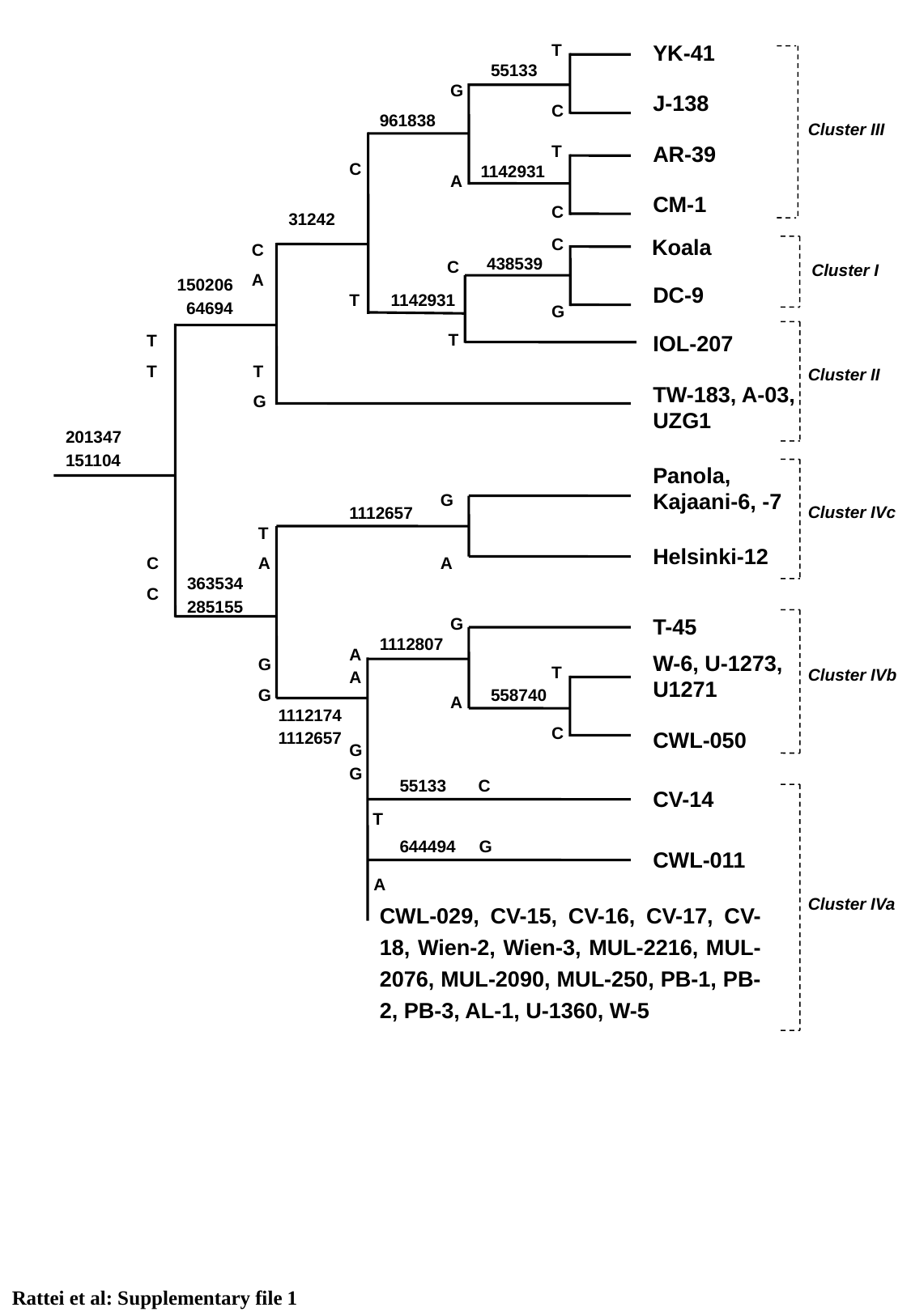

T
YK-41
55133
G
J-138
C
961838
Cluster III
T
AR-39
C
1142931
A
CM-1
C
31242
C
Koala
C
A
438539
C
Cluster I
150206
 64694
DC-9
T
1142931
G
T
T
T
IOL-207
T
G
Cluster II
TW-183, A-03, UZG1
201347
151104
Panola,
Kajaani-6, -7
G
Cluster IVc
1112657
T
A
Helsinki-12
C
C
A
363534
285155
G
T-45
1112807
A
A
W-6, U-1273, U1271
G
G
T
Cluster IVb
558740
A
1112174
1112657
C
CWL-050
G
G
55133
C
CV-14
T
644494
G
CWL-011
A
Cluster IVa
CWL-029, CV-15, CV-16, CV-17, CV-18, Wien-2, Wien-3, MUL-2216, MUL-2076, MUL-2090, MUL-250, PB-1, PB-2, PB-3, AL-1, U-1360, W-5
Rattei et al: Supplementary file 1
